# Molecular Insights into the Interaction between *Plasmodium falciparum* Apical Membrane Antigen 1 and an Invasion-Inhibitory Peptide

**DOI:** 10.1371/journal.pone.0109674

**Published:** 2014-10-24

**Authors:** Geqing Wang, Christopher A. MacRaild, Biswaranjan Mohanty, Mehdi Mobli, Nathan P. Cowieson, Robin F. Anders, Jamie S. Simpson, Sheena McGowan, Raymond S. Norton, Martin J. Scanlon

**Affiliations:** 1 Medicinal Chemistry, Monash Institute of Pharmaceutical Sciences, Monash University, Parkville, Victoria, Australia; 2 Australian Research Council Centre of Excellence for Coherent X-ray Science, Monash University, Parkville, Victoria, Australia; 3 Centre for Advanced Imaging, University of Queensland, St Lucia, Queensland, Australia; 4 Australian Synchrotron, Clayton, Victoria, Australia; 5 Department of Biochemistry, La Trobe University, Bundoora, Victoria, Australia; 6 Department of Biochemistry and Molecular Biology, Monash University, Clayton, Victoria, Australia; INSERM, France

## Abstract

Apical membrane antigen 1 (AMA1) of the human malaria parasite *Plasmodium falciparum* has been implicated in invasion of the host erythrocyte. It interacts with malarial rhoptry neck (RON) proteins in the moving junction that forms between the host cell and the invading parasite. Agents that block this interaction inhibit invasion and may serve as promising leads for anti-malarial drug development. The invasion-inhibitory peptide R1 binds to a hydrophobic cleft on AMA1, which is an attractive target site for small molecules that block parasite invasion. In this work, truncation and mutational analyses show that Phe5-Phe9, Phe12 and Arg15 in R1 are the most important residues for high affinity binding to AMA1. These residues interact with two well-defined binding hot spots on AMA1. Computational solvent mapping reveals that one of these hot spots is suitable for small molecule targeting. We also confirm that R1 in solution binds to AMA1 with 1∶1 stoichiometry and adopts a secondary structure consistent with the major form of R1 observed in the crystal structure of the complex. Our results provide a basis for designing high affinity inhibitors of the AMA1-RON2 interaction.

## Introduction

Malaria is a deadly infectious disease caused by protozoan parasites of the genus *Plasmodium*. The recently released World Malaria Report estimated that malarial parasites infected over 200 million people worldwide causing 627,000 deaths in 2012 [1]. The increasing incidence of drug resistance, absence of an effective vaccine and lack of diversity amongst current compounds in development renders this ancient disease an ongoing global health problem [2]. Novel anti-malaria therapeutic approaches are urgently required to confront these challenges.

The blood stage of *Plasmodium* infection is the major cause of the clinical symptoms of malaria and the mechanism of erythrocyte invasion is highly conserved in all apicomplexan parasites [3]. Therefore, proteins involved in this process have been actively pursued as targets for both vaccine and drug development. Apical membrane antigen 1 (AMA1), an integral membrane protein that is highly conserved throughout the phylum Apicomplexa, represents one of these protein targets [2]. The initiation of merozoite invasion is marked by formation of the moving junction (MJ), a ring-like protein structure, between the merozoite and the erythrocyte [4]. In our current understanding of the structure and function of the MJ, AMA1 presents a conserved hydrophobic cleft that interacts with rhoptry neck protein 2 (RON2) [5]. This interaction is essential to the formation of the junction, which commits the parasite to invade [4], [6]. Both AMA1 and RON2 are provided by the parasite to enable an active invasion mechanism [7]. AMA1 is initially stored in the parasite micronemes and subsequently translocated to the merozoite surface before invasion, while RON2 is secreted from the parasite rhoptry and transferred to the erythrocyte surface prior to invasion [8]–[10]. The essential role of AMA1 in host cell invasion has been questioned recently by genetic studies, which showed AMA1-depleted parasites can still form a functional MJ [11], [12]. As such, the specific role of AMA1 in host cell invasion remains a matter of debate [13], [14], but it is clear that inhibition of the AMA1-RON2 interaction by various agents effectively disrupts invasion and validates AMA1 as a viable therapeutic target [2], [15], [16]. Specifically, antibodies raised against AMA1 can inhibit invasion by binding to the hydrophobic cleft [17]–[19], although the inhibition is usually strain-specific [20]. Consistent with these observations, AMA1 evolves under strong selective pressure from the host immune system [21], [22], and loops surrounding the hydrophobic cleft are polymorphic [23]. Nonetheless, the AMA1-RON2 interaction is highly conserved. In addition, the interaction between AMA1 and RON2 can be inhibited by peptides. One such peptide, R1, was identified from a random peptide library using phage-display [24], [25]. R1 showed a high binding affinity for 3D7 *Pf*AMA1 (*K*_D_∼0.08 µM) [26] and spans the full-length of the hydrophobic cleft [27], [28]. Comparison with the structure of a complex between AMA1 and a peptide derived from RON2 reveals that the two peptides occupy the same region of AMA1 and exhibit structural mimicry [27]. Consistent with these structural studies, R1 can effectively inhibit erythrocyte invasion by malaria parasites *in vitro* [25], [28]. Although the inhibition is strain-specific, it has been demonstrated that *N*-methyl modification of R1 broadened its strain specificity [26].

It is evident from the current data that effective targeting of AMA1 from multiple strains requires inhibitors whose interaction is mediated by conserved residues within the hydrophobic cleft, which bind AMA1 without making extensive contact with polymorphic residues. It is likely that this goal will be more easily realized by using smaller molecules as inhibitors. We and others have recently reported the identification of small molecules that bind to AMA1, with the goal of developing these molecules into therapeutically useful antimalarials [15], [29]. A common problem faced in small molecule inhibitor design is difficulty in improving the binding affinity and specificity of screening “hits”. Identification of binding “hot spots”, *i.e.* the subset of residues at the binding interface that contribute most of the free energy to high affinity binding [30], provides important information to guide the design of high-affinity ligands. This is especially critical for targeting protein-protein interactions (PPIs) [31]. As R1 has high binding affinity and makes extensive interactions with the hydrophobic cleft of AMA1, characterization of the AMA1-R1 interaction provides valuable insights into the key interactions that contribute to binding. Indeed, there are many examples showing that small molecule inhibitors can be designed that mimic the interaction of a peptide with a protein target [32]–[37]. In the current study we have undertaken a detailed biophysical characterization of the interaction of R1 with AMA1 and used computational solvent mapping to identify hot spots at the binding interface. Collectively our data provide a rational basis for designing high-affinity inhibitors of AMA1-RON2 interaction.

## Materials and Methods

### Expression and purification of AMA1

Domain I+II of 3D7 *Pf*AMA1 (residue 104–442) was expressed, purified and refolded as described [29]. The folding of the purified protein was assessed by monitoring its binding affinity and stoichiometry to R1 using surface plasmon resonance and recording a 1D ^1^H spectrum, which is characterized in the correctly-folded material by the presence of several upfield-shifted methyl protons (Figure S1 in File S1). Randomly fractionally deuterated (f-^2^H) AMA1 was prepared by growth of expression cultures in 100% ^2^H_2_O/M9 minimal medium supplemented with ^14^NH_4_Cl (1 g/L) and protonated ^12^C-D-glucose (10 g/L). The high-cell-density method was implemented to achieve high protein yield as described in [38]. The hexahistidine (His_6_) tag of AMA1 was cleaved by tobacco etch virus (TEV) protease in a ratio of 0.02 mg TEV per mg fusion protein in phosphate buffer, pH 8.0 at 4°C for 24 h [39]. The resultant protein was purified on a linear gradient of 0–500 mM NaCl using HiTrap QFF column chromatography (GE healthcare) and dialyzed against 20 mM ammonium bicarbonate solution at 4°C over 2 days before it was lyophilized.

### DNA manipulation, expression and purification of R1

R1 peptide was produced recombinantly as an enterokinase-cleavable fusion to thioredoxin. An insert encoding DDDDKVFAEFLPLFSKFGSRMHILK was ligated into pET32a (Novagen) at KpnI/NcoI and transformed into *Escherichia coli* BL21 (DE3). The f-^2^H, u-^13^C, ^15^N-labelled R1 fusion was expressed in 100% ^2^H_2_O/M9 minimal medium supplemented with ^15^NH_4_Cl (1 g/L) and protonated ^13^C_6_-glucose (4 g/L) using the high-cell-density method as described in [38]. The cells were harvested by centrifugation at 5,000 g for 20 min and resuspended in lysis/wash buffer (20 mM Tris-HCl pH 8, 20 mM imidazole, 200 mM NaCl). The cells were lysed by sonication and the supernatants were recovered by centrifugation at 12,000 g for 30 min at 4°C. The His_6_-tagged R1 fusion in the soluble fraction was purified on a linear gradient of 45–500 mM imidazole by HisTrap column chromatography (GE healthcare). Fractions were analyzed by SDS-PAGE and those containing a band consistent with the expected size of the R1 fusion (∼20 kDa) were pooled and dialyzed against enterokinase cleavage buffer (20 mM Tris-HCl pH 7.4, 50 mM NaCl, 2 mM CaCl_2_, 1 mM EDTA) overnight at 4°C. The fusion protein was then incubated with recombinant enterokinase (Novagen) in a ratio of 0.5 units enterokinase per mg fusion protein at room temperature for 21 h. The sample was then filtered through a 0.22 µm membrane (Millipore, Merck) and purified using HiTrap QFF column chromatography using a gradient of 0–500 mM NaCl in a buffer of 20 mM Tris-HCl pH 8. R1 peptide was finally purified by prep-RP-HPLC using a Phenomenex Luna 5 u C18 column (100×10 mm). The identity and purity ( >95%) were confirmed by liquid chromatography mass spectrometry (LCMS) (Figure S2 in File S1). About 1 mg of f-^2^H, u-^15^N, ^13^C-labelled R1 was produced from 0.7 L of minimal medium. ^2^H incorporation was ∼72%, ^15^N incorporation was ∼90%, and ^13^C incorporation was ∼95%.

### Synthetic R1 analogues

Truncated and mutant R1 peptides used in the SPR study were synthesized by Mimotopes (Melbourne, Australia) with purity >90% and all were *N*-terminally acetylated and *C*-terminally amidated.

### NMR sample preparation

NMR samples were prepared in a buffer consisting of 20 mM sodium phosphate pH 7, 1 mM EDTA, 0.01% (w/v) sodium azide, 0.2% (w/v) Complete protease inhibitor cocktail (Roche), 50 mM Arg, 50 mM Glu and 6% (v/v) ^2^H_2_O unless noted otherwise. For the NMR study of free R1, two samples of u-^13^C, ^15^N-labelled R1 at a concentration of 0.4 mM were prepared at pH 5 and pH 7, respectively. To study the AMA1-R1 complex, lyophilized f-^2^H-labelled AMA1 was added to a sample of f-^2^H, u-^13^C, ^15^N-labelled R1 to give final concentrations of AMA1 and R1 of 320 and 300 µM, respectively. Based on the measured *K*_D_ of R1 for AMA1, >90% of the peptide should be bound to AMA1 under these conditions.

### NMR spectroscopy

NMR experiments for free R1 were performed at 5°C or 40°C at a ^1^H frequency of either 500 MHz or 600 MHz on Bruker Avance spectrometers equipped with a TXI-cryoprobe. Chemical shift assignments were made using the following experiments: 2D ^1^H-^15^N-HSQC, ^1^H-^13^C-HSQC and 3D triple-resonance experiments including HNCACB, CBCA(CO)NH, HBHA(CO)NH and HCCH-TOCSY. All spectra were processed using NMRPipe [40] and analyzed with CARA [41]. All NMR experiments for the AMA1-R1 complex were performed at 40°C in a 5 mm Shigemi tube. The backbone H^N^, C^α^, and N resonances of f-^2^H, u-^13^C, ^15^N-R1 bound to f-^2^H-AMA1_104–442_ were assigned using 2D ^1^H-^15^N-TROSY HSQC/conventional ^1^H-^15^N-HSQC, 3D TROSY-HNCA and TROSY-HN(CO)CA. The 3D TROSY-HNCA was acquired on a Bruker DRX-900 spectrometer equipped with a cryoprobe. Non-uniform sampling was utilized during acquisition, with sampling points chosen randomly from a probability distribution matching the signal decay, as described previously [42]. The spectra were re-constructed using the maximum entropy method with automated parameter selection using the Rowland NMR toolkit [43]. A ^13^C(F_2_)-^1^H(F_3_) plane of the 3D TROSY-HN(CO)CA was acquired on a Bruker Avance 600 MHz spectrometer. The data were processed using NMRPipe or Topspin 3.0 (Bruker-Biospin) and analyzed with CARA. Chemical shifts are reported relative to sodium 2,2-dimethyl-2-silapentane-5-sulfonate (DSS).

### Surface plasmon resonance binding analysis

A Biacore T200 biosensor instrument was used to measure the affinity of the interaction of peptides with 3D7 *Pf*AMA1_104–442_. AMA1 was immobilized onto a CM5 chip as described [29]. Surface plasmon resonance (SPR) experiments were performed at 25°C using HBS-EP (10 mM HEPES, 150 mM NaCl, 3.4 mM EDTA, and 0.05% surfactant P20, pH 7.4) as the running buffer either with (alanine scanning mutagenesis study) or without (truncation study) 1% DMSO. All peptide samples were prepared in the appropriate running buffer. To generate the peptide binding data, peptide at concentrations ranging from 10 nM to 10 µM was injected over immobilized AMA1 at a constant flow rate of 60 µL/min for 1.5 min; peptide dissociation was monitored by flowing running buffer at 60 µL/min for 5 min. The surface was regenerated after each cycle by injecting glycine/HCl at pH 2.0. Sensorgrams were first zeroed on the y-axis and then x-aligned at the start of the injection. Bulk refractive index changes were eliminated by subtracting the reference flow cell responses. For kinetic analysis, *k_a_* and *k_d_* were determined from the processed data sets by globally fitting to a 1∶1 binding model. For rapidly associating/dissociating truncated peptides, *K*_D_ was determined by fitting to a steady-state affinity model using a fixed R_max_ that was calculated based on the response of R1_5–16_.

### Analytical size exclusion chromatography (SEC)

Analytical SEC was performed on a Superdex 75 HR 10/30 column (column dimension 1.0×30 cm, column volume 23.6 mL) at room temperature. Samples (100 µl) containing AMA1 (200 µM) with or without R1 peptide (250 µM) were injected onto the column, which was pre-equilibrated with 20 mM sodium phosphate pH 7. Samples were prepared in NMR buffer (20 mM sodium phosphate pH 7, 1 mM EDTA, 0.01% (w/v) sodium azide, 0.2% (w/v) Complete protease inhibitor cocktail (Roche), 50 mM Arg and 50 mM Glu). The flow rate was maintained at 0.5 mL/min and the elution was monitored by measurement of UV absorbance at 280 nM (A_280_).

### Small angle X-ray scattering (SAXS)

SAXS measurements were made at the SAXS-WAXS beamline of the Australian Synchrotron, Melbourne, Australia. For each SAXS measurement, 10×1 s exposures were measured and averaged together after verifying that there was no evidence of radiation damage (systematic change in the shape of the scattering curves as a function of exposure time). During data collection the sample was flowed through a 1.5 mm quartz capillary at a rate of 4 µl/sec to further control for radiation damage. Measurements were performed on a dilution series of AMA1 alone from 3.3 to 0.14 mg/ml in NMR buffer and AMA1+R1 (ratio of 1∶1.15) from 3.0 to 0.19 mg/ml in the same buffer. Some concentration-dependent aggregation was observed at protein concentrations above 1 mg/ml as evidenced by increases in Rg and disproportionate increases in I(0) (data not shown). The SAXS data used in this study were from protein at 0.5 mg/ml for AMA1 and 0.75 mg/ml for AMA1:R1. Dilution of the protein below these concentrations did not result in changes to the shape of the scattering curve and calculated molecular weights at these concentrations were consistent with monomeric protein. The molecular weights of the scattering species were estimated from the total forward scatter of the SAXS measurements that were normalised by comparison to water scatter and with reference to the measured protein concentrations. Partial specific volume and scattering length density were calculated using the program MULCh [44]. The monomeric state of the protein was inferred by comparison of the theoretical molecular weight of the protein sequence with the calculated molecular weight from the SAXS experiment. A 1.6 m camera was used with an X-ray energy of 11 keV giving a Q range from 0.01 to 0.5 Å^−1^. Data were collected on a Pilatus 1M detector (Dektris) and averaging of images, subtraction of blanks and radial integration was performed using the beamline control software ScatterBrain (Australian Synchrotron). Measurements were made at 25°C. Calculation of scattering intensities from molecular models was done using CRYSOL [45]. Radius of gyration (Rg), total forward scatter (I(0)) and P(r) functions were derived using the automated functions in PRIMUS [46] and without manual intervention.

### Computational mapping of binding hot spots

FTMAP was employed to map the binding hot spots of AMA1 (http://ftmap.bu.edu/) [47] using the AMA1 structures with PDB ID 3SRJ and 2Z8V, which were downloaded from the Protein Data Bank [18], [27]. All ligands and water molecules were removed before mapping. FTMAP searched the global surface of AMA1 with a library of 16 small organic molecules (ethanol, isopropanol, isobutanol, acetone, acetaldehyde, dimethyl ether, cyclohexane, ethane, acetonitrile, urea, methylamine, phenol, benzaldehyde, benzene, acetamide and *N,N*-dimethylformamide). The small molecule probes have different hydrophobicity and hydrogen bonding capability. FTMAP employs a fast Fourier transform correlation approach to efficiently sample billions of protein-probe complexes [48]. The 2000 most favourable docked positions of each probe were energy-minimized and clustered. The six clusters with the lowest average free energy were selected for each probe. The clusters of different probes were further clustered into consensus sites (CSs) based on the distance between the cluster centres. The details of the FTMAP algorithm are described in [48].

### Accession Numbers

Chemical shift assignments for free R1 (pH 5, 40°C) and AMA1-bound R1 (pH 7, 40°C) have been deposited in BMRB under accession codes 19864 and 25134, respectively.

## Results and Discussion

### Truncation of the R1 peptide

**Figure 1:**
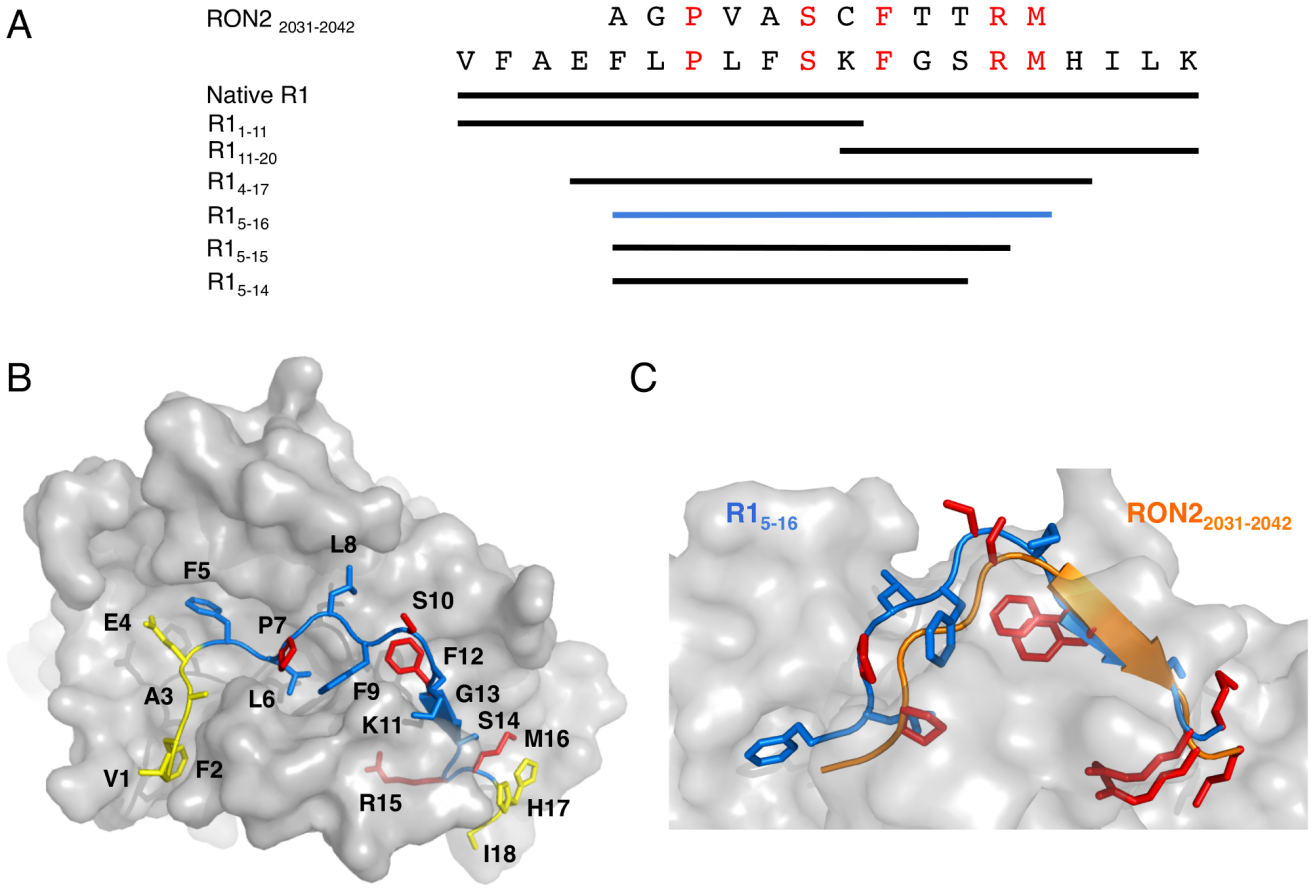
Identification of the minimal binding construct of R1 peptide. A. Amino acid sequences of *Pf*RON2_2031–2042_, native R1 and truncated peptides. Residues that are conserved between R1 and RON2 are highlighted in red. B. Co-crystal structure of *Pf*AMA1 bound to R1 peptide (PDB ID: 3SRJ, [27]). AMA1 is presented as a grey surface; R1 is presented as a cartoon (the minimal binding construct Phe5-Met16 is shown in blue, Val1-Glu4 and His17-Ile18 are in yellow). Side chains of the conserved residues are highlighted in red. The minor form of R1 is omitted in this structure. C. Structural comparison of R1_5–16_ (blue) and *Pf*RON2_2031–2042_ (orange) bound to AMA1. The structure of the R1 peptide bound to *Pf*AMA1 (PDB ID: 3SRJ) superimposed onto the co-crystal structure of *Pf*AMA1-*Pf*RON2 (PDB ID: 3ZWZ, [27]). Only Phe5-Met16 of R1 and Ala2031-Met2042 of *Pf*RON2 are shown for clarity.

**Figure 2:**
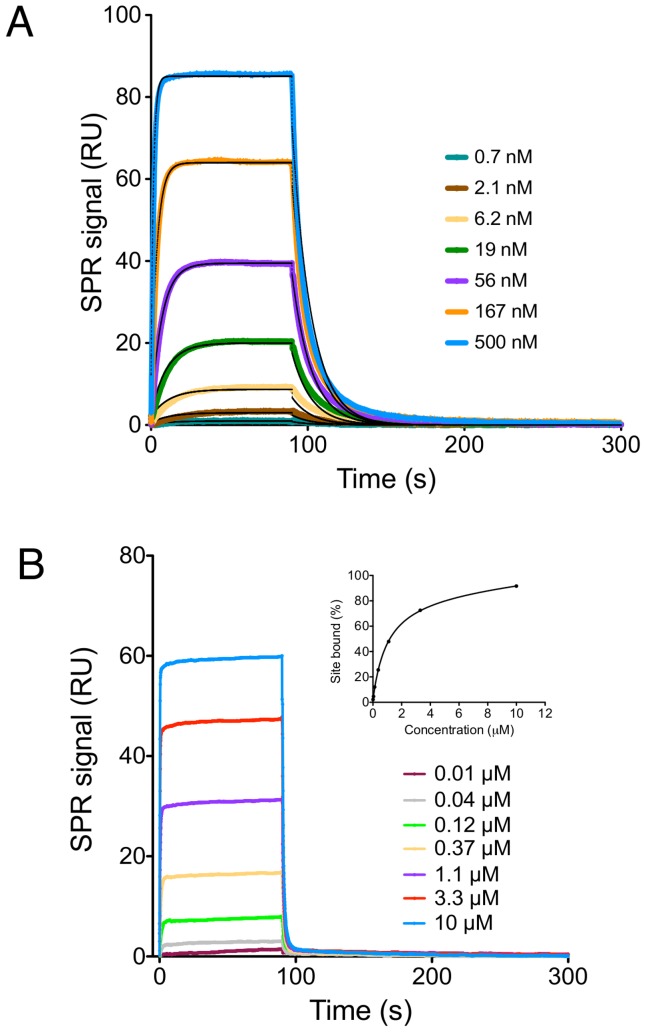
SPR analysis of peptides binding to immobilized 3D7 *Pf*AMA1_104–442_. A series of concentrations, as indicated in sensorgrams, of native R1 (panel A) and truncated R1_5–16_ (panel B) was injected over the AMA1-immobilized surface. *k*_a_ and *k*_d_ of native R1 were determined by globally fitting to a 1∶1 binding model. The apparent equilibrium dissociation constants *K*_D_ for other peptides were determined using a steady-state affinity model and are given in Table 1.

We sought to identify key residues in the interaction of R1 with AMA1. Firstly, in order to define the minimal R1 construct that retains high binding affinity for 3D7 *Pf*AMA1, a series of truncated R1 analogues (Figure 1A) was synthesized and screened by SPR. Kinetic analysis of data generated for native R1 binding to AMA1 produced a *K*_D_ of 0.11 µM by globally fitting to a 1∶1 binding model (Figure 2A), which is consistent with the reported value (∼0.08 µM) [26]. Our previous mutagenesis studies had shown that Phe5, Pro7, Leu8 and Phe9 of R1 were essential for high affinity binding of R1 to AMA1 [49]. This conclusion was supported by the current data, in which the truncated R1_11–20_ showed no binding to AMA1 up to 10 µM (Table 1). Interestingly, R1_1–11_ containing the Phe5-Phe9 segment also displayed no detectable binding to AMA1 up to 10 µM (Table 1), implying that the residues Phe5-Phe9 are necessary but not sufficient for interaction with AMA1, and that other key residues are required to facilitate high affinity binding.

**Table 1:** Equilibrium dissociation constants (*K*_D_) determined by SPR for the interaction of truncated R1 with 3D7 *Pf*AMA1_104–442_.

Peptide	*K*_D_ (µM)^a^
Native R1	0.11
R1_1–11_	No binding^b^
R1_11–20_	No binding^b^
R1_4–17_	0.88
R1_5–16_	0.99
R1_5–15_	4.6
R1_5–14_	No binding^b^

a Equilibrium dissociation constants (*K*_D_) were estimated using a kinetic algorithm or a steady-state affinity algorithm available within the Biacore T200 evaluation program.

SPR was performed in HBS-EP running buffer (no DMSO) at 25°C.

b No binding event was observed up to a peptide concentration of 10 µM.

To test this hypothesis, R1_4–17_ and R1_5–16_ peptides were synthesized and their binding affinities were measured by SPR. Since both of these truncated mutants showed fast association and dissociation kinetics (Figure 2B), a steady-state affinity model was used to fit the data, producing *K*_D_ values of 0.88 µM for R1_4–17_ and 0.99 µM for R1_5–16_. Although the binding affinity of the peptides was reduced nearly 10-fold relative to native R1, the fact that both peptides retain *K*_D_<1 µM suggests that Val1-Glu4 and His17-Lys20 do not contribute substantially to high affinity binding with AMA1 (Figure 1B). Further truncation to the 11-residue peptide R1_5–15_ resulted in a further ∼5-fold reduction in *K*_D_ to 4.6 µM. However truncation of this peptide by deletion of Arg15 to generate the 10-residue R1_5–14_ completely abolished measurable binding (Table 1), indicating that Arg15 is essential for high-affinity binding of R1 to AMA1. This result is consistent with the co-crystal structure of AMA1 bound to R1, in which Arg15 of R1 is bound in a pocket at one end of the hydrophobic cleft of AMA1 (Figure 1B), where it forms four hydrogen bonds and is the residue that contributes the largest proportion to the buried surface in the interface [27]. Therefore, R1_5–16_ was determined to be the minimal construct that retained relatively high binding affinity (∼1 µM) to AMA1. This segment of R1 displays remarkable structural similarity to the Ala2031-Met2042 segment of *Pf*RON2, with an RMSD of 1.2 Å over the twelve C^α^ positions in their respective structures, implying that the high affinity of R1_5–16_ originates from direct mimicry of the natural ligand RON2 as previously suggested (Figure 1C) [27].

### Alanine-scanning mutagenesis of the R1 peptide

To identify the key interacting residues of R1_5–16_, alanine-scanning mutagenesis was performed and the binding affinities of the mutants were determined by SPR (Table 2). It was necessary to include 1% DMSO (v/v) in the running buffer for this SPR study to maintain the solubility of all of the peptides. This resulted in a small drop in the affinity of the interaction with R1_5–16_ (Table 2). Previous ELISA assays on four single-point mutants of R1 had demonstrated that mutation of Pro7 abrogated R1 binding, while mutation of Phe5, Leu8 and Phe9 each resulted in 7.5-, 86- and >140-fold reductions in affinity relative to the full-length peptide, respectively [49]. In the current SPR study, substitution of Pro7 to Ala resulted in a 35-fold reduction in affinity for AMA1 relative to R1_5–16_, indicating that Pro7 is one of the residues that are crucial for high affinity binding. In the crystal structure of the AMA1-R1 complex [27], Pro7 does not make any direct contact with AMA1, suggesting that it may play a structural role to maintain the adjacent residues in an appropriate conformation for binding (Figure 1B). In addition, substitution of Leu6 to Ala caused a 33-fold reduction in affinity for AMA1 relative to R1_5–16_ (Table 2). Leu6 makes interactions with a cluster of five Tyr residues in AMA1 (Tyr142, Tyr 175, Tyr234, Tyr 236 and Tyr 251). Importantly, Tyr 251 is highly conserved in *Plasmodium* species and has been shown to be essential for AMA1-RON2 interactions [4], [50]. Combining current and previous data [49], every residue in the hydrophobic sequence Phe5-Phe9 contributes significantly to AMA1 binding. In the crystal structure of the AMA1-R1 complex, Phe5-Phe9 interacts with a well-defined pocket on one end of the hydrophobic cleft (Figure 1B). All of the above suggest that the pocket is a binding hot spot on AMA1 and potentially an attractive target site.

**Table 2:** Equilibrium dissociation constants (*K*_D_) determined by SPR for the interaction of R1_5–16_ mutants with 3D7 *Pf*AMA1_104–442_.

Peptide	Sequence	*K*_D_ (µM)^a^
R1_5–16_	Ac-FLPLFSKFGSRM-NH_2_	1.8±0.04^b^
R1_5–16_ L6A	Ac-F**A**PLFSKFGSRM-NH_2_	60±12
R1_5–16_ P7A	Ac-FL**A**LFSKFGSRM-NH_2_	61±25
R1_5–16_ S10A	Ac-FLPLF**A**KFGSRM-NH_2_	4.8±0.8
R1_5–16_ K11A	Ac-FLPLFS**A**FGSRM-NH_2_	27±2.1
R1_5–16_ F12A	Ac-FLPLFSK**A**GSRM-NH_2_	87±5.9
R1_5–16_ G13A	Ac-FLPLFSKF**A**SRM-NH_2_	39±3.9
R1_5–16_ S14A	Ac-FLPLFSKFG**A**RM-NH_2_	3.3±0.3
R1_5–16_ R15A	Ac-FLPLFSKFGS**A**M-NH_2_	>100
R1_5–16_ M16A	Ac-FLPLFSKFGSR**A**-NH_2_	2.7±0.2

a Equilibrium dissociation constants (*K*_D_) were estimated using a steady-state affinity algorithm available within the Biacore T200 evaluation program.

The data are expressed as mean ± standard error of the means (SEM). All experiments were conducted on at least three independent occasions.

b SPR for R1_5–16_ and its mutants was performed in the presence of 1% DMSO (v/v) in HBS-EP running buffer at 25°C.

A substantial drop in affinity (48-fold relative to R1_5–16_) was observed for Ala mutation at Phe12. In the crystal structure, the aromatic ring of Phe12 interacts with two of the key Tyr residues Tyr236 and Tyr251 in the hot spot. In addition, it interacts with Phe183, which was previously identified as a key residue for *Pf*AMA1-*Pf*RON2 interaction [27]. Ala mutation at Phe2038 of RON2 (equivalent to Phe12 of R1, Figure 1A) abolished the binding of RON2 to AMA1 [27]. A 15-fold reduction in affinity relative to R1_5–16_ was observed for the Lys11Ala mutant. This may be caused by disruption of the H-bonds that are observed in the structure between the Lys side chain and Asp227 of AMA1. Mutation of Gly13 resulted in a 21-fold reduction in binding affinity relative to R1_5–16_. Since Gly13 interacts with AMA1 through backbone residues only, this loss in affinity may be the result of conformational changes or steric clashes introduced by the mutation. In contrast to the residues discussed above, individual replacements of Ser10, Ser14 and Met16 with Ala resulted in less than 3-fold reductions in affinity, implying that these residues do not contribute significantly to the binding affinity for AMA1. In the crystal structure of the complex, the side chains of these residues are pointing away from the hydrophobic cleft of AMA1 such that mutation to Ala can be accommodated (Figure 1B) [27].

Consistent with both the truncation studies and the crystal structure, substitution of Arg15 to Ala resulted in largest reduction in affinity ( >60-fold relative to R1_5–16_) (Table 2). The importance of the Arg residue at this position is similar to the case with a peptide derived from RON2, where substitution of Arg2041 of RON2 (equivalent to Arg15 of R1, Figure 1A) to Ala abolished the binding to AMA1 [27]. In the structures of their complexes, Arg2041 of *Pf*RON2 interacts with the same pocket of AMA1 as Arg15 of R1 and is the residue that contributes most of the buried surface in the interface (Figure 1C). In addition to R1 and RON2, antibodies IF9 and IgNAR, which bind with high affinity to AMA1, also have either Arg or Lys residues that fit into the same pocket in the hydrophobic cleft in their respective structures [27]. Together, these data confirm that this “Arg pocket” is a binding hot spot on AMA1, which may serve as a pivotal anchor point for RON2 binding and an attractive site for inhibiting the AMA1-RON2 interaction.

### Backbone resonance assignments of the AMA1-bound R1 peptide

**Figure 3:**
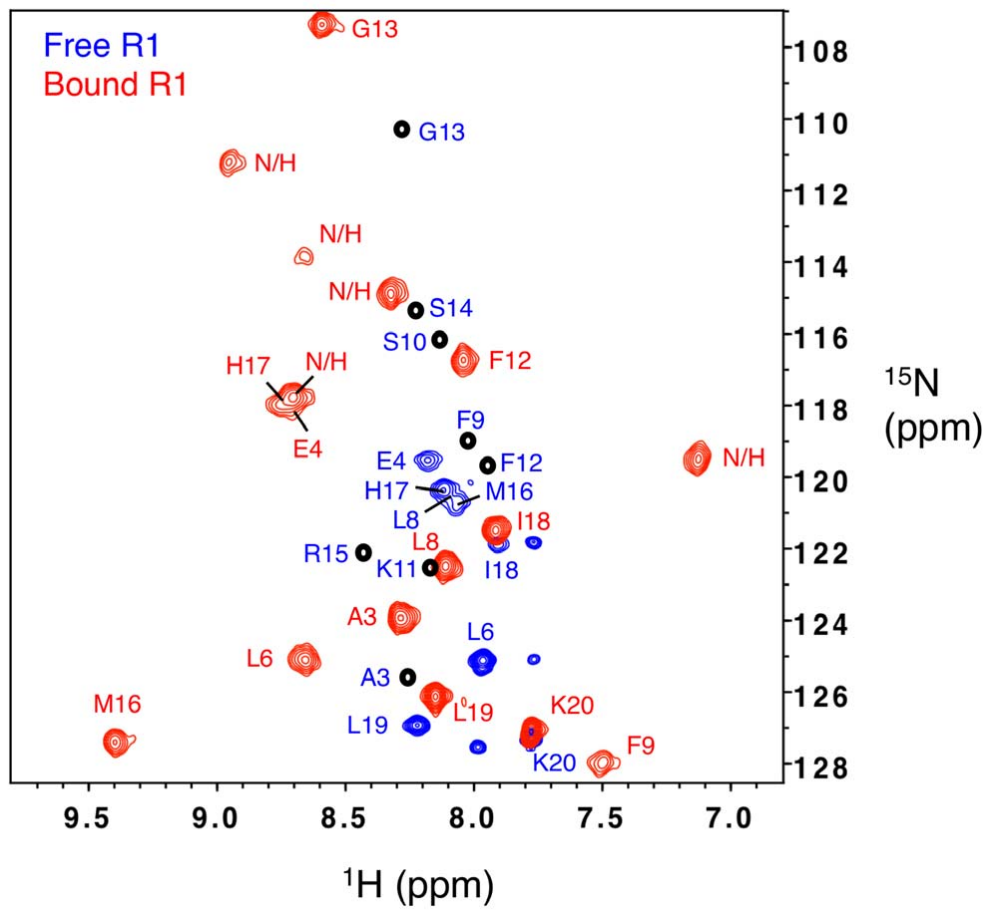
Comparison of the ^1^H-^15^N-HSQC spectra of f-^2^H, u-^13^C, ^15^N-labelled R1 in the absence (blue) and presence (red) of a saturating concentration of fractionally deuterated 3D7 *Pf*AMA1_104–442_ at pH 7 and 40°C. Some amide resonances (Ala3, Phe9, Ser10, Lys11, Phe12, Gly13, Ser14 and Arg15) of free R1 were broadened beyond detection at pH 7 and 40°C and their predicted resonances are indicated as black circles in the spectrum (prediction was made as described in the text). N/H indicates unassigned amide resonances of bound R1.

The crystal structure of the AMA1-R1 complex revealed a somewhat unexpected 2∶1 binding stoichiometry, which contrasted with the 1∶1 binding observed previously by SPR and ITC studies [27]. To resolve this apparent anomaly, we investigated the AMA1-R1 interaction by solution NMR spectroscopy. A recombinant protein expression system was established to produce uniformly (u-) ^13^C, ^15^N-labelled R1 peptide (Figure S2–4 in File S1). Backbone resonance assignments for free u-^13^C, ^15^N-labelled R1 were obtained at pH 7 and 5°C using standard triple-resonance experiments. For the free peptide it was necessary to record the spectrum at a lower temperature as several peaks were not observed at 40°C (which was found to be the optimum temperature for recording spectra of the complex), presumably due to their rapid exchange with water (Figure 3). To enable comparison of the free and bound states, amide chemical shifts for free R1 were extrapolated to 40°C by recording a series of ^1^H-^15^N-HSQC spectra at increasing temperatures and calculating the temperature dependence of the amide resonances (Table S1 in File S1).

A sample of fractionally deuterated (f-^2^H), u-^13^C, ^15^N-labelled R1 with excess f-^2^H-labelled AMA1 was prepared for backbone assignment of bound R1. To ensure that all the R1 peptide was in the bound form, samples with different ratios of the R1:AMA1 were also prepared. It was found that when the R1:AMA1 ratio was >1, the ^1^H-^15^N-HSQC spectrum contained two sets of peaks corresponding to free R1 and bound R1, respectively (Figure S5 in File S1). This indicates that R1 is in slow exchange with AMA1, which is consistent with its high binding affinity. If two peptides were bound to one AMA1 molecule as shown in the crystal structure, this would either give rise to a second set of bound signals in the spectrum or lead to perturbation of the chemical shifts of free R1 in the spectrum recorded with a sub-stoichiometric amount of AMA1; however, no additional peaks or chemical shift perturbations corresponding to a second bound state of R1 were observed. Thus the NMR result supports the 1∶1 binding stoichiometry indicated by our SPR data and previous ITC data [27].

**Figure 4:**
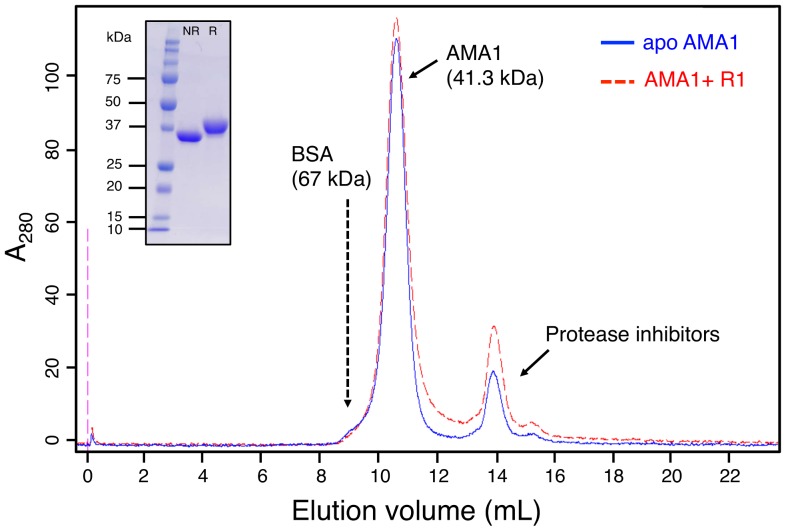
Elution profile of AMA1 in the absence (blue) and presence (red) of R1 peptide on an analytical size exclusion column. The elution time of bovine serum albumin (BSA) was determined for the size exclusion column that was used to elute AMA1 (Superdex 75 HR 10/30, column dimension 1.0×30 cm, column volume 23.6 mL). Both apo AMA1 (200 µM) and R1-bound AMA1 (200 µM AMA1+250 µM R1) showed a similar elution profile. The peak eluting at 10.5 mL is consistent with monomeric AMA1 (MW 41.3 kDa when His tag is not cleaved). SDS-PAGE (inset) confirms only one protein band corresponding to AMA1 is present. NR = non-reducing, R = reducing. The peak at ∼14 mL results from the addition of Complete protease inhibitor cocktail (Roche) to the buffer (Figure S8 in File S1), and was also verified by mass spectroscopy.

**Figure 5:**
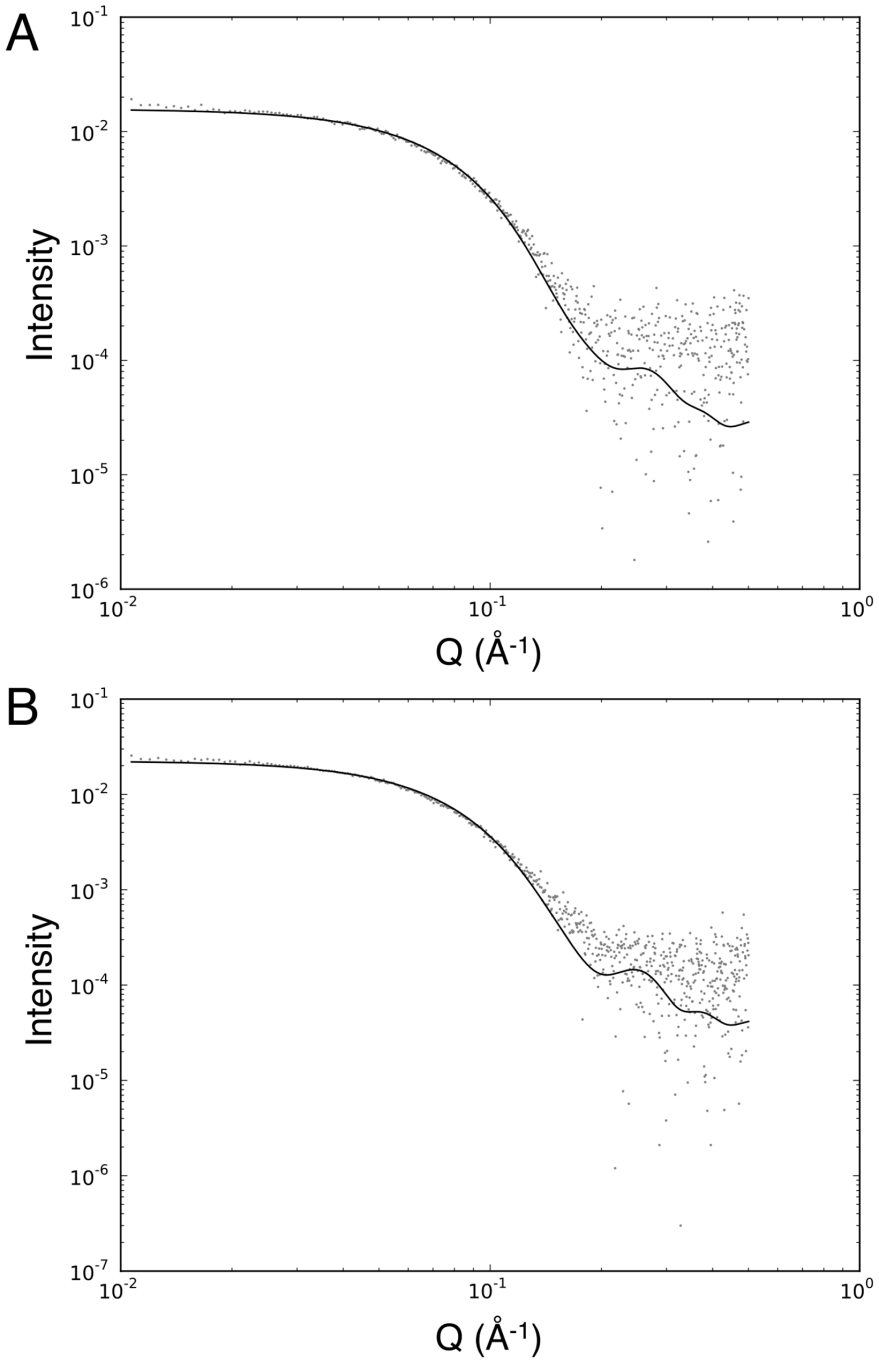
SAXS analysis of AMA1 alone and AMA1 in the presence of R1. (A) AMA1 scattering data fitted to the crystal structure of AMA1 (PDB 1Z40) (Chi-square = 0.61). (B) AMA1+R1 scattering data fitted to the crystal structure of AMA1 bound to R1 (PDB 3SRJ) (Chi-square = 0.72). Q is the momentum transfer vector.

R1 is a 20-residue peptide containing a single proline and has a free *N*-terminal amine. Therefore, a total of 18 peaks were expected in the ^1^H-^15^N-HSQC spectrum of bound R1. Of these, 17 were observed for the bound R1 peptide at pH 7 and 40°C (Figure 3), although the peak intensities were non-uniform across the spectrum. Both analytical size-exclusion chromatography (Figure 4) and small-angle X-ray scattering (SAXS) data (Figure 5) indicate that AMA1 interacts with R1 as a monomer, with no evidence for higher order oligomers of protein. The monomeric state is inferred from the SAXS data both from the goodness of fit to the monomeric crystal structures (Figure 5) and from the molecular weight calculated from total forward scattering (37 kDa for apo AMA1 and 41 kDa for AMA1+R1. These values compare to theoretical molecular weights of 41.3 and 43.7 kDa respectively). This suggests that the poor sensitivity of certain residues in the NMR spectra is most likely caused by local conformational exchange in the complex that results in significant broadening for the peaks of affected residues. This effect also resulted in poor sensitivity in 3D experiments and hindered full backbone assignment. Through careful analysis of both TROSY-HNCA and TROSY-HN(CO)CA spectra (Figure S6,7 in File S1), 12 out of 18 expected amide resonances and 15 out of 20 expected C^α^ resonances were assigned (Table S2 in File S1).

### Structural analysis of the AMA1-bound R1 peptide

Free R1 displayed narrow chemical shift dispersion in the proton dimension (7.7 ppm–8.5 ppm) of the ^1^H-^15^N-HSQC, consistent with the largely disordered structure in solution that has been observed previously (Figure 3 and Figure S3–4 in File S1) [25]. Upon binding to AMA1 the ^1^H-^15^N-HSQC spectrum of the peptide showed broader chemical shift dispersion in the proton dimension (7.0–9.5 ppm), consistent with the peptide assuming a more ordered conformation. The crystal structure of R1 bound to AMA1 identified two R1 peptides bound to AMA1, which were described as the “major” and “minor” states [27]. However, only one set of amide peaks was observed in the ^1^H-^15^N-HSQC for bound R1 (Figure 3).

**Figure 6:**
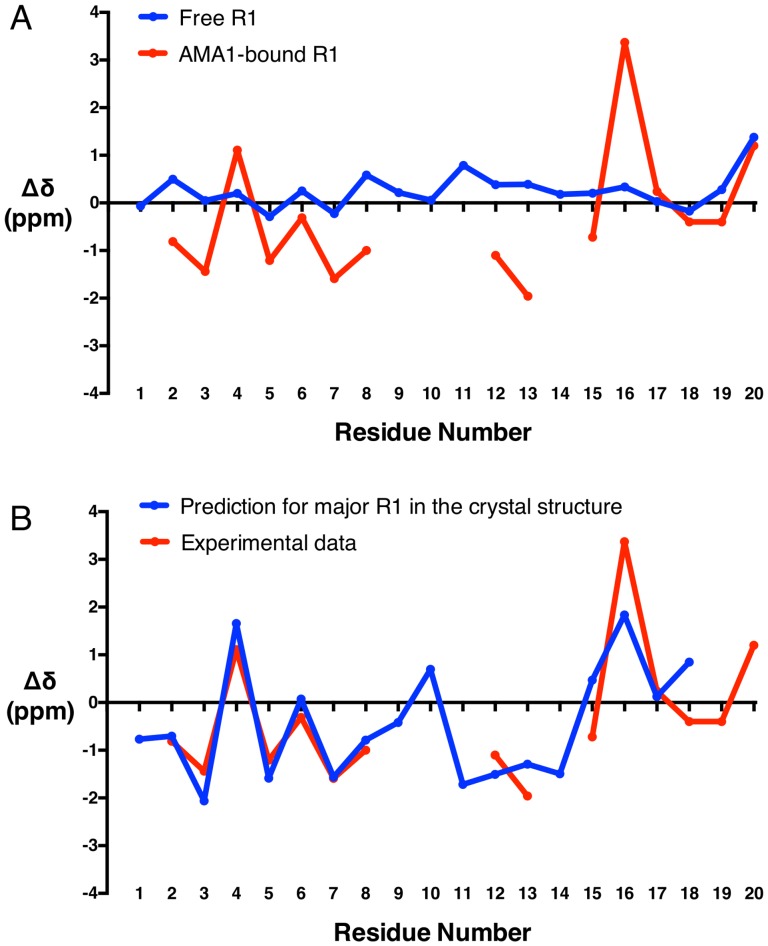
NMR C^α^ secondary chemical shifts. A. The C^α^ secondary shifts for free (blue) and AMA1-bound (red) R1 at pH 7 and 40°C. B. Comparison of the experimentally determined C^α^ secondary shifts of bound R1 (red) and C^α^ secondary shifts predicted for the major form of R1 bound to AMA1 in the crystal structure (Chain C, PDB ID: 3SRJ) using SHIFTX2 (blue) [53].

As R1 “minor binder” makes several contacts with R1 “major binder” in the crystal structure, we sought to evaluate the possible structural changes of bound R1 for the 1∶1 binding stoichiometry that is observed in solution. Due to the poor sensitivity in 3D experiments, we were not able to solve the solution structure of bound R1 and make direct comparison with the crystal structure. Instead, we probed the secondary structure of bound R1 based on a limited set of assigned C^α^ chemical shifts and compared that with the secondary structure of bound R1 in the crystal structure. The deviation of the C^α^ chemical shifts in R1 relative to their random coil values [51] (secondary shifts, Δδ) was calculated as these are correlated with the polypeptide backbone torsion angles φ and ψ [52] and can be used to predict the secondary structure of AMA1-bound R1 in solution. The secondary shifts of both free and bound R1 are plotted in Figure 6A. The secondary shifts for free R1 are close to zero. In contrast, bound R1 showed larger secondary shifts. Although the C^α^ chemical shift of Ser14 remained unassigned, Phe12 and Gly13 showed reasonably strong negative secondary shifts (Phe12 and Gly13 <−1), which is consistent with the presence of extended β-structure in Phe12-Gly13-Ser14 as revealed by the crystal structure of major R1 bound to AMA1 [27]. The *C*-terminal residues His17-Lys20 of bound R1 showed nearly identical C^α^ secondary shifts to those of free R1, which is consistent with this region being flexible in solution and suggests that these residues may not be directly involved in the interaction with AMA1.

To further evaluate secondary structure similarity between bound R1 in solution and in the crystal, a comparison was made of C^α^ chemical shifts, which were determined experimentally for bound R1 in solution and predicted for the major form of R1 in the crystal structure. The predictions for R1 in the crystal structure (Chain C, PDB ID: 3SRJ) were performed using SHIFTX2 [53]. The predicted results are plotted as secondary shifts in Figure 6B. Although the C^α^ chemical shifts of some residues were unassigned or missing, the secondary C^α^ shifts of the experimental data and the crystal structure prediction were strikingly similar. C^α^ chemical shifts were also predicted for the minor R1 in the crystal structure, but the correlation between the predicted data and experimental data was much poorer (correlation coefficient for Glu4-Leu8 of minor R1 = 0.85; correlation coefficient for Glu4-Leu8 of major R1 = 0.99; Figure S9 in File S1). The distinction between the two binding modes was equally unambiguous when chemical shifts were predicted by SPARTA+ instead of SHIFTX2 (Figure S10 in File S1). This suggests that the secondary structure of AMA1-bound R1 in solution is similar to that of the major R1 in the crystal structure. Taken together, the stoichiometry observed in the SPR data we report here, the previous ITC data and comparison of the experimental and predicted NMR data suggest that the minor R1 conformation was most likely an artifact due to the high concentration of R1 peptide used in the crystallographic study.

### Computational solvent mapping of AMA1

**Figure 7:**
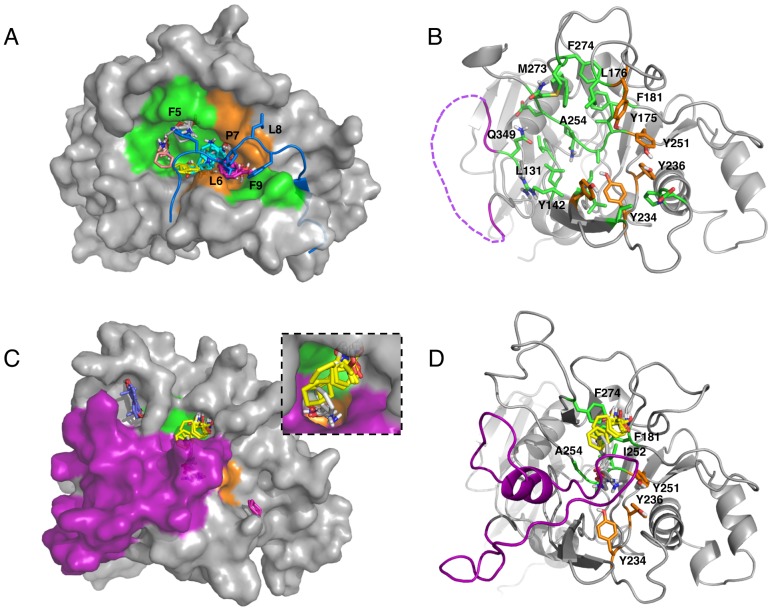
Computational solvent mapping of AMA1 using FTMAP. (A) Mapping results for R1-bound 3D7 *Pf*AMA1 (grey, PDB ID: 3SRJ). R1 peptides and water were removed prior to mapping. The five largest consensus sites, CS1 (cyan, 21 probe clusters), CS2 (magenta, 17 probe clusters), CS3 (yellow, 13 probe clusters), CS4 (salmon, 10 probe clusters) and CS5 (grey, 9 probe clusters) are located in a large hydrophobic pocket that binds to the Phe5-Phe9 segment of R1 peptide. The position of the R1 peptide in the crystal structure is shown for reference (blue). Phe5-Phe9 side chains are displayed as sticks and labelled individually as shown in the figure. The surface of the pocket is coloured according to side chain colours in panel B. (B) The residues that interact with small molecule probe clusters in the pocket are shown as sticks (green/orange). A cluster of five Tyr residues is highlighted in orange. Broken purple line indicates the displaced domain II loop in the R1-bound conformation. (C) Mapping results for IgNAR-bound 3D7 *Pf*AMA1 (grey, PDB ID: 2Z8V). IgNAR and water were removed prior to mapping. Two consensus sites CS3 (yellow, 14 probe clusters) and CS5 (grey, 9 probe clusters) are located in a domain II loop-protected pocket. The surface of the protein is coloured according to colour scheme in panel D. An inset highlights probes that fit into a deep, narrow pocket. (D) Some key interacting residues (highlighted as green/orange sticks), which bind probes when the domain II loop is displaced (panel B), are still solvent accessible to small molecule organic probes when the pocket is partially protected by the domain II loop (purple).

Using truncation and mutagenesis of R1 peptide, we have identified two binding hot spots on the AMA1 surface that contribute to high affinity of R1 binding. To further assess the capacity of these hot spots to effectively bind small organic molecules, we employed FTMAP, a fragment-based computational solvent mapping algorithm [47]. FTMAP searched the global surface of the AMA1 with a library of 16 small molecule probes that vary in hydrophobicity and hydrogen bonding capability [48]. The regions that bind to probe clusters are designated consensus sites (CS) in FTMAP and the site that binds the highest number of probe clusters is identified as the most druggable. We performed the initial mapping on the structure of 3D7 *Pf*AMA1 co-crystallized with R1 (PDB ID: 3SRJ). Prior to mapping, the bound ligands and water were removed. The five largest consensus sites were located in the same pocket, which was also identified from SPR analysis as a hot spot for binding Phe5-Phe9 of R1, implicating it as a prospective pocket for small molecule targeting (Figure 7A). The most notable features of the pocket are its hydrophobicity and conservation. The pocket is flanked at one end by a cluster of five Tyr residues (Tyr142, Tyr175, Tyr234, Tyr236 and Tyr251) and at the other end by Leu176, Ala254, Met273 and Phe274 (Figure 7B). Tyr251 is highly conserved across *Plasmodium* species and essential for AMA1-RON2 interactions [4], [50]. All the other residues that form the pocket, except Tyr175, are also highly conserved in all known *P. falciparum* sequences [2].

Of the probe clusters identified, CS2 (magenta, 17 probe clusters) overlaps well with Leu6 of R1 and partially with Phe9 of R1; CS5 (grey, 9 probe clusters) overlaps well with Phe5 of R1 (Figure 7A). Probes in CS2 favour hydrogen bonding to phenol hydroxyl groups of Tyr234, Tyr236 and Tyr251. More importantly, the largest consensus site CS1 (cyan, 21 probe clusters) and CS3 (yellow, 13 probe clusters), which are located on the base of the pocket, revealed the key interactions that are in addition to those formed by the Phe5-Phe9 segment of R1. Probes in CS1 and CS3 make additional interactions with Leu131, Arg143, Leu144, Pro145, Ala253 and Gln255. CS4 (salmon, 10 probe clusters) extends one end of the pocket by interacting with Val129, Gln256 and Gln349 (Figure 7B). Probes in CS4 favour hydrogen bonding to the amide group of Gln349. The mapping results presented here suggest that this hot spot, which interacts with Phe5-Phe9 of R1, is druggable and effectively binds various small organic molecules. Identification of additional key interactions in the hot spot is potentially useful in the development of small molecule inhibitors.

The Phe5-Phe9-interacting hot spot is partially protected by the domain II loop, which is displaced by the binding of R1 and RON2. Part of this hot spot was identified previously as a pocket for small molecule targeting, although it has been suggested that the domain II loop may limit small molecule binding at this site [54]. We have shown that ∼420 Å^3^ solvent-accessible volume of this pocket is still available for small molecule binding when the domain II loop is not displaced [2]. To further address this issue, we performed mapping on the structure of 3D7 *Pf*AMA1 co-crystallized with antibody IgNAR (PDB ID: 2Z8V). This is the only AMA1 structure that has a complete description of the domain II loop [18]. IgNAR binds to a region distant from the hot spot and does not induce any significant changes in the structure of AMA1 (C^α^ RMSD of 0.34 Å between IgNAR-bound and apo AMA1). Our mapping for 2Z8V results showed that two consensus sites CS3 (yellow, 14 probe clusters) and CS5 (grey, 9 probe clusters) are located in the domain II loop-protected pocket (Figure 7C). Importantly, several of the key residues, which bind Phe5-Phe9 of R1 or small molecule probes when the domain II loop is displaced, are still involved in the formation of the loop-protected pocket and remain accessible to small organic molecules (Phe181, Tyr234, Tyr236, Tyr251, Ile252, Ala253, Ala254 and Phe274; Figure 7D). All these residues are highly conserved in *P. falciparum* and their side chain conformations remain almost unchanged when the domain II loop is displaced.

Notably, no consensus sites were found in the Arg pocket in either AMA1 structure. One possible explanation could be that the Arg pocket is relatively small and has a polar surface area. Although the interactions mediated in the Arg pocket are absolutely crucial for R1/RON2 binding to AMA1, it may be more difficult to identify suitable small molecules to access and interact with this site in isolation.

## Conclusions

Using SPR and NMR spectroscopy we have validated that R1 binds to AMA1 in solution with 1∶1 stoichiometry, as suggested by previous ITC data [27], and adopts a secondary structure consistent with the major form of R1 observed in the crystal structure of the complex. The minor form of R1 in the crystal structure was not observed in solution and is likely to be a crystallographic artifact. The truncation and mutational studies for R1 presented here have identified several key AMA1-interacting residues scattered along the peptide. Amongst these key residues, the hydrophobic segment Phe5-Leu6-Pro7-Leu8-Phe9, residues Phe12 and Arg15 are those that contribute most to the AMA1 binding affinity. They interact with two distinct binding hot spots, which are located at the two ends of the hydrophobic cleft of AMA1. Both of the pockets are highly conserved across the *P. falciparum* strains and likely to be suitable for designing broad-spectrum AMA1 inhibitors. The “Arg pocket” at one end of the cleft mediates key interactions of several known inhibitory agents, although fragment-based computational solvent mapping on AMA1 suggests that it may be a difficult site to target with small organic molecules because of its small surface area and polar nature. Mimicking the Arg side chain using peptidomimetics based on R1 or RON2 might be a more productive approach to target this important pocket. In contrast, mapping results showed that the Phe5-Phe9-interacting hot spot is druggable and identified key AMA1 residues for small molecule targeting. Our results provide a basis for designing novel high affinity inhibitors of AMA1-RON2 interaction that are effective against the majority of *Pf*AMA1 genotypes.

## Supporting Information

File S1 **Supporting information.** Result S1, Backbone resonance assignments for AMA1-bound R1. Figure S1, 1D ^1^H spectrum of 3D7 *Pf*AMA1_104–442_ in 20 mM sodium phosphate pH 7, acquired at 600 MHz and 40°C. Figure S2, The purity ( >95%) (A) and mass (B) of f-^2^H, u-^13^C, ^15^N-labelled R1 peptide were verified using LCMS. Figure S3, ^1^H-^15^N-HSQC spectrum of 0.4 mM u-^13^C,^15^N-labelled R1 at pH 5 and 40°C. Figure S4, Comparison of deviation of ^1^H chemical shifts (H^N^, top panel; H^α^, bottom panel) from random coil values for previously reported synthetic R1 at pH 4.5, 5°C and ^13^C, ^15^N-labelled R1 at pH 7, 40°C (this study). Figure S5, ^1^H-^15^N-HSQC spectra of 0.3 mM f-^2^H, u-^13^C, ^15^N-labelled R1 in the presence of increasing concentration of f-^2^H-AMA1. Figure S6, Strip plot of the 3D TROSY-HNCA spectrum of the f-^2^H, u-^13^C, ^15^N-labelled R1-f-^2^H-labelled AMA1 complex. Figure S7, The ^13^C(F_2_)-^1^H(F_3_) plane of the 3D TROSY-HN(CO)CA spectrum of the ^2^H,^13^C,^15^N-labelled R1-^2^H-labelled AMA1 complex. Figure S8, Elution profile of NMR buffer on an analytical size exclusion column. Figure S9, C^α^ secondary shifts predicted for the minor form of R1 in the crystal structure using SHIFTX2. Figure S10, C^α^ secondary shifts predicted for R1 in the crystal structure using SPARTA+. Table S1, Chemical shifts of free R1 at pH 7 and 40°C. Table S2, Chemical shifts of AMA1-bound R1 at pH 7 and 40°C.
(DOCX)
