# Supplementary material for: Molecular Insights into the Interaction between Plasmodium falciparum Apical Membrane Antigen 1 and an Invasion-Inhibitory Peptide
Source: PLoS One. 2014 Oct 24;9(10):e109674. doi: 10.1371/journal.pone.0109674 (PMC4208761; doi:10.1371/journal.pone.0109674)
Supplement: File S1 — Supporting information. Result S1, Backbone resonance assignments for AMA1-bound R1. Figure S1, 1D 1H spectrum of 3D7 PfAMA1104–442 in 20 mM sodium phosphate pH 7, acquired at 600 MHz and 40°C. Figure S2, The purity ( >95%) (A) and mass (B) of f-2H, u-13C, 15N-labelled R1 peptide were verified using LCMS. Figure S3, 1H-15N-HSQC spectrum of 0.4 mM u-13C,15N-labelled R1 at pH 5 and 40°C. Figure S4, Comparison of deviation of 1H chemical shifts (HN, top panel; Hα, bottom panel) from random coil values for previously reported synthetic R1 at pH 4.5, 5°C and 13C, 15N-labelled R1 at pH 7, 40°C (this study). Figure S5, 1H-15N-HSQC spectra of 0.3 mM f-2H, u-13C, 15N-labelled R1 in the presence of increasing concentration of f-2H-AMA1. Figure S6, Strip plot of the 3D TROSY-HNCA spectrum of the f-2H, u-13C, 15N-labelled R1-f-2H-labelled AMA1 complex. Figure S7, The 13C(F2)-1H(F3) plane of the 3D TROSY-HN(CO)CA spectrum of the 2H,13C,15N-labelled R1-2H-labelled AMA1 complex. Figure S8, Elution profile of NMR buffer on an analytical size exclusion column. Figure S9, Cα secondary shifts predicted for the minor form of R1 in the crystal structure using SHIFTX2. Figure S10, Cα secondary shifts predicted for R1 in the crystal structure using SPARTA+. Table S1, Chemical shifts of free R1 at pH 7 and 40°C. Table S2, Chemical shifts of AMA1-bound R1 at pH 7 and 40°C. (DOCX) [file pone.0109674.s001.docx]

**File S1- Supporting information**

**Molecular Insights into the Interaction between *Plasmodium* *falciparum* Apical Membrane Antigen 1 and an Invasion-Inhibitory Peptide**

Geqing Wang,^1^ Christopher A. MacRaild,^1^ Biswaranjan Mohanty,^1,2^ Mehdi Mobli,^3^ Nathan P. Cowieson,^4^ Robin F. Anders,^5^ Jamie S. Simpson,^1^ Sheena McGowan,^6^ Raymond S. Norton,^1^* Martin J. Scanlon^1,2^*

**1** Medicinal Chemistry, Monash Institute of Pharmaceutical Sciences, Monash University, Parkville, Victoria, Australia

**2** Australian Research Council Centre of Excellence for Coherent X-ray Science, Monash University, Parkville, Victoria, Australia

**3** Centre for Advanced Imaging, University of Queensland, St Lucia, Queensland, Australia

**4** Australian Synchrotron, Clayton, Victoria, Australia

**5** Department of Biochemistry, La Trobe University, Bundoora, Victoria, Australia

**6** Department of Biochemistry and Molecular Biology, Monash University, Clayton, Victoria, Australia.

* To whom correspondence should be addressed:

Martin J. Scanlon, Medicinal Chemistry, Monash Institute of Pharmaceutical Sciences, Parkville, Victoria, Australia

Tel: (+61 3) 9903 9540; Fax: (+61 3) 9903 9582; Email: martin.scanlon@monash.edu

Raymond S. Norton, Medicinal Chemistry, Monash Institute of Pharmaceutical Sciences, Parkville, Victoria, Australia

Tel: (+61 3) 9903 9167; Fax: (+61 3) 9903 9582; Email: ray.norton@monash.edu

**Supplementary results**

**Result S1. Backbone resonance assignments for AMA1-bound R1**

A sample of uniformly ^13^C, ^15^N-labelled R1 with excess unlabelled AMA1 was initially prepared for backbone assignment of bound R1. However, only 6 of the 18 expected amide resonances were observed in ^1^H-^15^N-TROSY spectra at temperatures as high as 40 °C. The peak broadening or intensity losses were presumably due to a combination of the fast T_2_ relaxation of the high-molecular-mass complex (>40 kDa), low concentration and the relatively fast rate of amide proton exchange at the experiment pH of 7.4. To overcome these difficulties, both R1 and AMA1 were fractionally deuterated (f-^2^H) to reduce the T_2_ relaxation rate [1]. In addition, Arg (50 mM) and Glu (50 mM) were included in the NMR buffer, which enabled us to increase the concentration of AMA1 in the complex from 200 μM to 320 μM and reduce the pH from 7.4 to 7 [2]. As a result, a sample of 300 μM f-^2^H, u-^13^C, ^15^N-labelled R1 with 320 μM f-^2^H AMA1 was prepared for backbone assignment of bound R1. 17 out of the 18 expected amide resonances were observed in ^1^H, ^15^N-HSQC/TROSY spectra of the bound R1 peptide at pH 7 and 40 °C. A 3D TROSY-HNCA was subsequently acquired at 900 MHz and 40 °C in order to obtain backbone assignments for R1. Only 9 residues showed both C^α^ and C^α-1^ peaks in the corresponding strip of the HNCA spectrum, whilst 3 strips contained a single C^α^ frequency and 5 residues were missing both their C^α^ and C^α-1^ peaks (Figure S6 in File S1). Since the sensitivity of the HN(CO)CA is expected to be higher than that of the HNCA for large proteins [3], a TROSY-HN(CO)CA was acquired in an attempt to observe more C^α-1^ peaks. Rather than record a full 3D HN(CO)CA experiment, we took advantage of the excellent dispersion of R1 amide resonances in the ^1^H-^15^N-HSQC and acquired only a single ^1^H-^13^C plane of the 3D TROSY-HN(CO)CA. This allowed a greater number of scans to be recorded to increase the sensitivity of the experiment and helped identify a C^α-1^ peak for an extra three residues (Figure S7 in File 1).

The strategy used to assign amide and C^α^ chemical shifts based on limited data set is described below. Each amide resonance of the bound R1 peptide in ^1^H-^15^N-TROSY was picked and assigned as a spin system. Assignment was guided by sequential connectivity as well as the reported average chemical shifts for different residue types in the BMRB database. The most intense peaks of bound R1 were observed for the four *C*-terminal residues. Both C^α^ and C^α-1^ cross peaks were observed in the TROSY-HNCA data for Lys20, Leu19, Ile18, His17 and residues Met16-Lys20 were thereby unambiguously assigned. Their C^α^ chemical shifts were consistent with average BMRB database values. Although both C^α^ (in HNCA) and C^α-1^ peaks (in HN(CO)CA) were observed for Met16, no signals were observed in either spectrum that could be assigned to the spin system of the preceding residue Arg15. It is likely that Arg15 is either one of four spin systems that does not have either C^α^ or C^α-1^ peaks or that it is missing in the ^1^H-^15^N-HSQC; therefore the amide chemical shift of Arg15 could not be assigned. Spin system (H^N^=8.60, C^α^=43.14, C^α-1^=56.6) and spin system (H^N^=8.27, C^α^=51.1, C^α-1^=56.9) were readily assigned to Gly13 and Ala3 due to their distinctive C^α^ chemical shifts among the residues in the peptide sequence. Based on connectivity to C^α^ frequencies observed in the HNCA strips assigned to Gly13 and Ala3, Phe12 and Glu4 were also sequentially assigned. However, no connectivity to other residues was possible due to the absence of correlated peaks in the spectra. Spin system (H^N^=8.12, C^α^=54.1, C^α-1^=62.0) showed a high C^α-1^ chemical shift. Based on the typical chemical shifts recorded in BMRB, the most likely preceding residues are Val1 (BMRB average C^α^=62.57), Pro7 (BMRB average C^α^=63.36) or Ile18 (BMRB average C^α^=61.68). Of these residues, Ile18 was unambiguously assigned based on sequential connectivity in the HNCA spectrum. Val1 can be ruled out since the C^α^ resonance observed at 54.1 ppm does not connect with the C^α-1^ cross peak at 56.9 ppm that is present in the HNCA strip assigned to Ala3. Therefore this spin system was tentatively assigned to Leu8. A correlation was observed in the 3D HN(CO)CA spectrum at 54.1 ppm, which was assigned as the C^α-1^ of Leu8 and enabled tentative assignment of the amide resonance of Phe9. No cross peaks were observed in the 3D HNCA experiment for this amide resonance. At this point, 11 out of the 17 observed residues were assigned. There was one additional spin system (H^N^=8.70, C^α^=52.8, C^α-1^=56.5), which showed both C^α^ and C^α-1^ peaks in the HNCA, but for which connectivity could not be established to any other assigned residue. Based on comparison of the observed C^α^ chemical shifts with average BMRB values and peptide sequence, this spin system was assigned to Leu6. Two peaks were observed in the HN(CO)CA spectrum at the amide frequency assigned to Phe12. Since only a single resonance was observed in the HNCA strip, it was not possible to unambiguously assign either peak as the C^α^ of Lys11 either based on connectivity or typical chemical shift. In summary, 12 amide resonances were assigned for Ala3, Glu4, Leu6, Leu8, Phe9, Phe12, Gly13, Met16, His17, Ile18, Leu19 and Lys20; 6 amide resonances were not assigned (Phe2, Phe5, Ser10, Lys11, Ser14, and Arg15). In addition, 15 C^α^ chemical shifts were assigned to Phe2, Ala3, Glu4, Phe5, Leu6, Pro7, Leu8, Phe12, Gly13, Arg15, Met16, His17, Ile18, Leu19 and Lys20. The assigned chemical shifts of bound R1 are listed in Table S2 in File S1.

**Supplementary figures**

**Figure S1.** **1D ^1^H spectrum of 3D7 *Pf*AMA1_104-442_ in 20 mM sodium phosphate pH 7, acquired at 600 MHz and 40 °C.** Excitation sculpting was used for water suppression. Red arrows indicate high field methyl proton resonances that are characteristic of the folded protein.


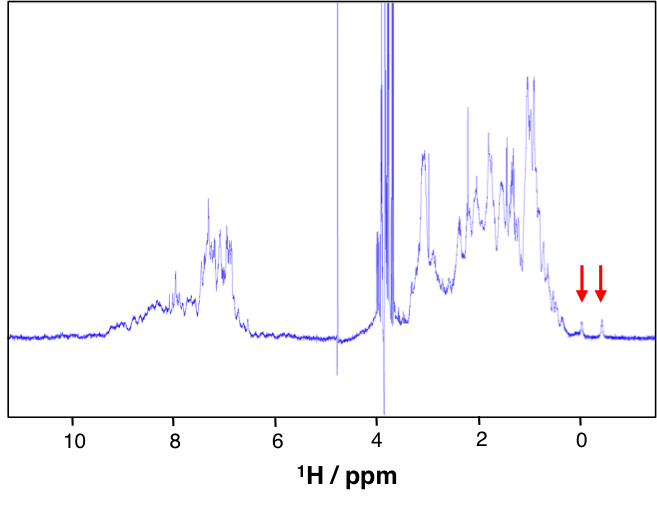


**Figure S2**. **The purity (>95%) (A) and mass (B) of f-^2^H, u-^13^C, ^15^N-labelled R1 peptide were verified using LCMS.**

**Figure S3.** **^1^H-^15^N-HSQC spectrum of 0.4 mM u-^13^C,^15^N-labelled R1 at pH 5 and 40 °C.** The spectrum was acquired at 500 MHz ^1^H frequency and 40 °C in a buffer of 20 mM sodium phosphate pH 5, 1 mM EDTA, 0.01% (w/v) sodium azide, 0.01% (w/v) Complete protease inhibitor cocktail (Roche) and 10% ^2^H_2_O. Data for chemical shift assignment (^H^N, N, C^α^, H^α^) of u-^13^C,^15^N-labelled R1 have been deposited to BMRB under code 19864.


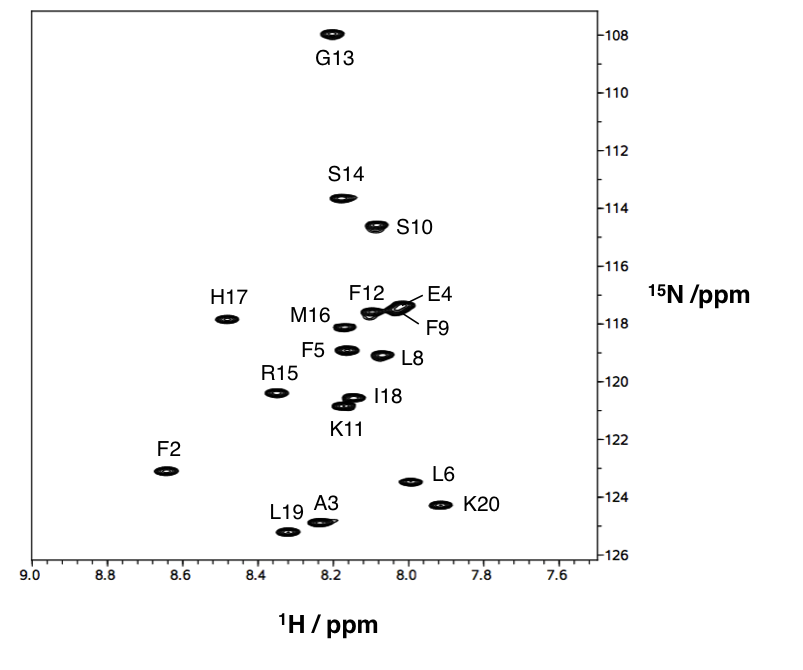


**H^N^**

**H^α^**

**Figure S4.** **Comparison of deviation of ^1^H chemical shifts (H^N^, top panel; H^α^, bottom panel) from random coil values for previously reported synthetic R1 at pH 4.5, 5 °C [4] and ^13^C,^15^N-labelled R1 at pH 7, 40 °C (this study).** The chemical shifts for ^13^C, ^15^N-labelled R1 are given in Table S1 in File S1. The random coil values used for calculation were reported by Merutka et al [5]. The chemical shifts deviation of H^N^ and H^α^ are very similar for both peptides allowing for the differences in pH and temperature.

Δδ / ppm

Δδ / ppm


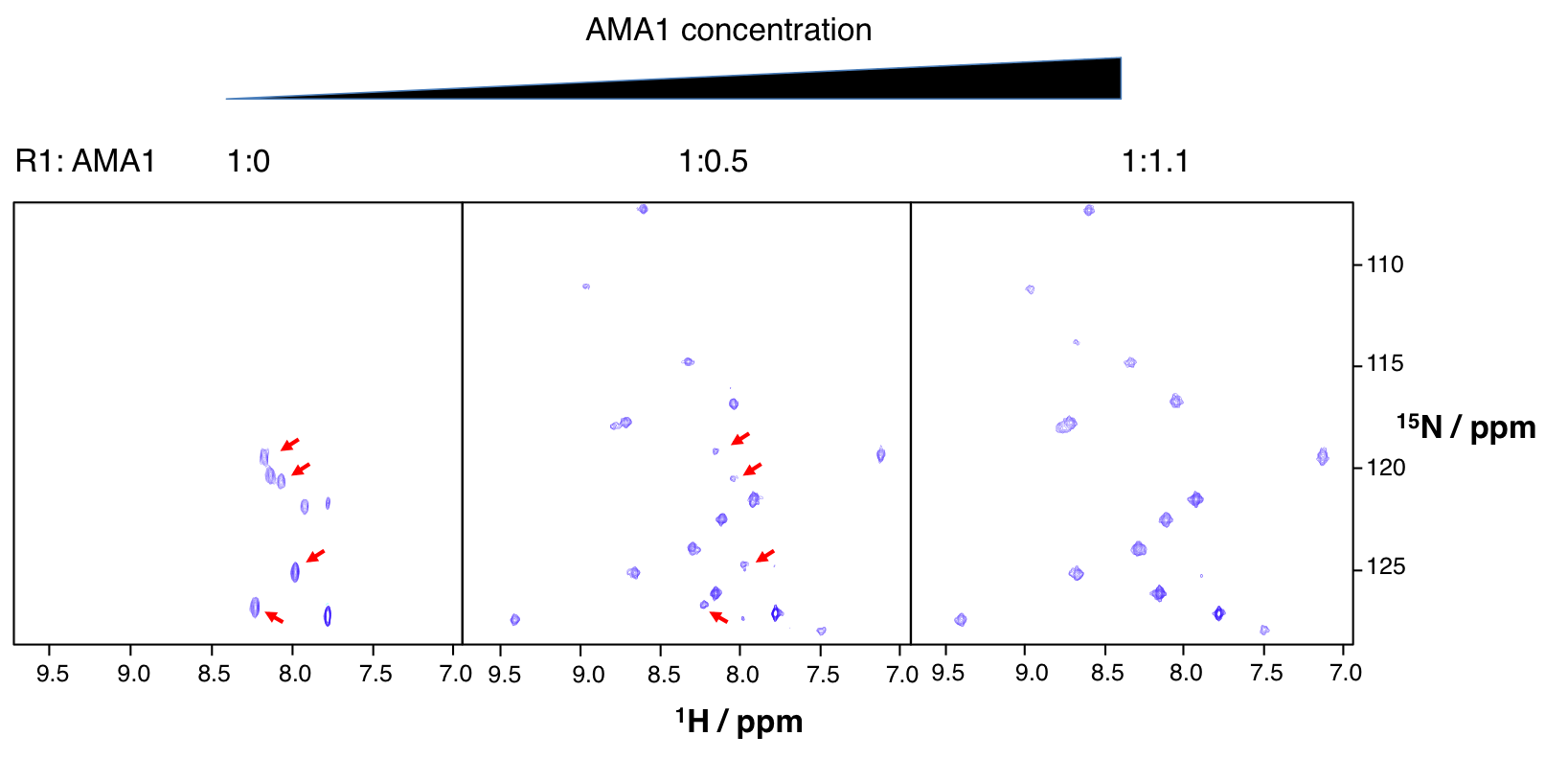


**Figure S5.** **^1^H-^15^N-HSQC spectra of 0.3 mM f-^2^H, u-^13^C, ^15^N-labelled R1 in the presence of increasing concentration of f-^2^H-AMA1.** Spectra were acquired at 600 MHz ^1^H frequency and 40 °C in a buffer of 20 mM sodium phosphate pH 7, 1 mM EDTA, 0.01% (w/v) sodium azide, 0.2% (w/v) Complete protease inhibitor cocktail (Roche), 50 mM Arg, 50 mM Glu and 6% ^2^H_2_O. Selected amide resonances from free R1 are indicated by red arrows.

**Figure S6.** **Strip plot of the 3D TROSY-HNCA spectrum of the f-^2^H, u-^13^C, ^15^N-labelled R1-f-^2^H-labelled AMA1 complex.** The spectrum was acquired at 900 MHz ^1^H frequency and 40 °C in a buffer consisting of 20 mM sodium phosphate pH 7, 1 mM EDTA, 0.01% (w/v) sodium azide, 0.2% (w/v) Complete protease inhibitor cocktail (Roche), 50 mM Arg, 50 mM Glu and 6% (v/v) ^2^H_2_O. C^α-1^ peaks that were identified from the TROSY-HN(CO)CA spectrum (Figure S7 in File S1) are marked in red.

**Figure S7.** **The ^13^C(F_2_)-^1^H(F_3_) plane of the 3D TROSY-HN(CO)CA spectrum of the ^2^H,^13^C,^15^N-labelled R1-^2^H-labelled AMA1 complex.** The spectrum was acquired at 600 MHz ^1^H frequency and 40 °C in a buffer consisting of 20 mM sodium phosphate pH 7, 1 mM EDTA, 0.01% (w/v) sodium azide, 0.2% (w/v) Complete protease inhibitor cocktail (Roche), 50 mM Arg, 50 mM Glu and 6% (v/v) ^2^H_2_O. C^α-1^ peaks that were not identified in the TROSY-HNCA spectrum (Figure S6 in File S1) are marked in red. Arrows indicate the presence of unassigned C^α-1^ peaks on the strips of particular amide proton chemical shift. Grey lines were used as visual guides. It should be noted that although two C^α-1^ peaks (one of them belongs to Glu4) have similar H^N^ chemical shift as indicated by arrow on the left, these two peaks were on the different HNCA planes and therefore can be unambiguously assigned.


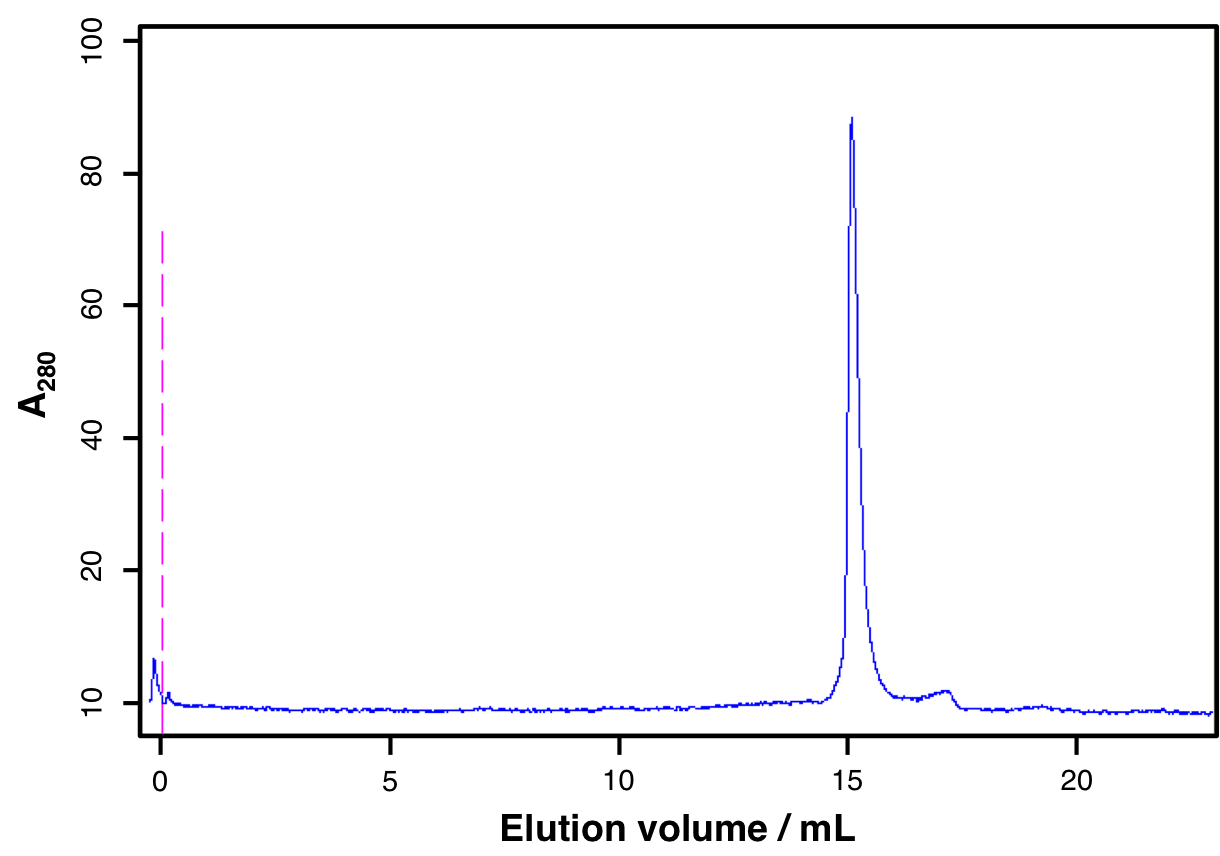


**Figure S8**. **Elution profile of NMR buffer on an analytical size exclusion column.** 200 μL NMR buffer (20 mM sodium phosphate pH 7, 1 mM EDTA, 0.01% (w/v) sodium azide, 0.2% (w/v) Complete protease inhibitor cocktail (Roche), 50 mM Arg and 50 mM Glu) was injected into Superdex 75 HR 10/30 column, which was pre-equilibrated with 20 mM sodium phosphate buffer, pH 7. The flow rate was maintained at 0.5 mL/min and the elution was monitored by measurement of UV absorbance at 280 nM (A_280_). The strongly absorbing peak originated from a component of complete protease inhibitor cocktail (Roche).

**Figure S9.** **C^α^ secondary shifts predicted for the minor form of R1 in the crystal structure using SHIFTX2.** C^α^ secondary shifts predicted for the minor form of R1 (green) were compared with C^α^ secondary shifts predicted for the major form of R1 (blue) bound to AMA1 in the crystal structure (PDB ID: 3SRJ) and experimentally determined C^α^ secondary shifts of bound R1 (red).

**Figure S10.** **C^α^ secondary shifts predicted for R1 in the crystal structure using SPARTA+ [6].** C^α^ secondary shifts predicted for the minor form of R1 (green) were compared with C^α^ secondary shifts predicted for the major form of R1 (blue) bound to AMA1 in the crystal structure (PDB ID: 3SRJ) and experimentally determined C^α^ secondary shifts of bound R1 (red). The correlation coefficient for experimental data *vs.* SPARTA+-predicted chemical shifts for major R1 (Glu4-Leu8) is 0.85, while for experimental data *vs.* SPARTA+-predicted chemical shifts for minor R1 (Glu4-Leu8) it is 0.57.

**Supplementary tables**

**Table S1**. **Chemical shifts of free R1 at pH 7 and 40 °C**

| **Residue** | **H^N^** | **N** | **C^α^** | **C^β^** | **H^α^** |
| --- | --- | --- | --- | --- | --- |
| **Val1** | - | - | 62.1 | 33.5 | 3.6 |
| **Phe2** ^c^ | ND | ND | 58.2 | 39.6 | 4.8 |
| **Ala3** ^b^ | 8.26 | 126.2 | 52.6 | 19.5 | 4.3 |
| **Glu4** ^a^ | 8.17 | 119.4 | 56.8 | 30.4 | 4.2 |
| **Phe5** ^c^ | ND | ND | 57.4 | 39.6 | 4.8 |
| **Leu6** ^a^ | 7.96 | 125.0 | 53.4 | 42.3 | 4.7 |
| **Pro7** | - | - | 63.4 | 31.8 | 4.4 |
| **Leu8** ^a^ | 8.05 | 120.6 | 55.7 | 42.3 | 4.3 |
| **Phe9** ^b^ | 8.00 | 119.0 | 57.9 | 39.5 | 4.7 |
| **Ser10** ^b^ | 8.10 | 116.2 | 58.4 | 63.9 | 4.5 |
| **Lys11** ^b^ | 8.14 | 122.6 | 57.0 | 32.8 | 4.3 |
| **Phe12** ^a^ | 8.01 | 120.1 | 58.1 | 39.7 | 4.7 |
| **Gly13** ^b^ | 8.22 | 109.8 | 45.5 | - | 4.0 |
| **Ser14** ^b^ | 8.20 | 115.6 | 58.5 | 63.9 | 4.6 |
| **Arg15** ^b^ | 8.40 | 122.1 | 56.2 | 30.6 | 4.5 |
| **Met16** ^a^ | 8.06 | 120.7 | 55.7 | 33.1 | 4.5 |
| **His17** ^a^ | 8.11 | 120.3 | 55.0 | 31.2 | 4.8 |
| **Ile18** ^a^ | 7.90 | 121.8 | 60.9 | 38.8 | 4.2 |
| **Leu19** ^a^ | 8.21 | 126.8 | 55.4 | 42.5 | 4.5 |
| **Lys20** ^a^ | 7.77 | 127.2 | 57.6 | 34.0 | 4.3 |

**^a^** Glu4, Leu6, Leu8, Phe12, Met16, His17, Ile18, Leu19 and Lys20 H^N^/N chemical shifts at pH 7 and 40 °C were obtained based on chemical shift assignments at pH 7 and 5 °C by tracking peak shifts at elevated temperatures. **^b^** Ala3, Phe9, Ser10, Lys11, Gly13, Ser14 and Arg15 amide resonances broadened beyond detection at higher temperatures, their H^N^/N chemical shifts were extrapolated to 40 °C by recording a series of ^1^H-^15^N-HSQC spectra over the temperature range 5 to 40 °C, at 5 °C intervals, and calculating the temperature dependence of the amide resonances using equation δH^N^ (T)=T_c_ × (T-5°C) + δH^N^ (5°C). T is temperature and T_c_ is temperature coefficient. **^c^** Phe2 and Phe5 amide resonances were not observed at pH 7 and 5 °C, therefore their HN/N chemical shifts at pH 7 and 40 °C were not determined (ND= not determined).

**Table S2**. **Chemical shifts of AMA1-bound R1 at pH 7 and 40 °C**

| **Residue** | **H^N^** | **N** | **C^α^** |
| --- | --- | --- | --- |
| **Val1** | - | - | ND |
| **Phe2** | ND ^a^ | ND | 56.9 |
| **Ala3** | 8.27 | 124.5 | 51.1 |
| **Glu4** | 8.73 | 118.8 | 57.7 |
| **Phe5** | ND | ND | 56.5 |
| **Leu6** | 8.70 | 125.8 | 52.8 |
| **Pro7** | - | - | 62.0 |
| **Leu8** | 8.12 | 123.1 | 54.1 |
| **Phe9** | 7.49 | 128.3 | ND |
| **Ser10** | ND | ND | ND |
| **Lys11** | ND | ND | ND |
| **Phe12** | 8.08 | 116.9 | 56.6 |
| **Gly13** | 8.60 | 107.9 | 43.1 |
| **Ser14** | ND | ND | ND |
| **Arg15** | ND | ND | 55.3 |
| **Met16** | 9.36 | 127.8 | 58.8 |
| **His17** | 8.76 | 118.4 | 55.2 |
| **Ile18** | 8.05 | 122.6 | 60.7 |
| **Leu19** | 8.20 | 126.8 | 54.7 |
| **Lys20** | 7.79 | 127.6 | 57.4 |

^a^ ND = not determined.

**Supplementary references**

[1] Gardner KH, Kay LE (1998) The use of ^2^H, ^13^C, ^15^N multidimensional NMR to study the structure and dynamics of proteins. Annu Rev Biophys Biomol Struct 27, 357-406.

[2] Golovanov AP, Hautbergue GM, Wilson SA, Lian LY (2004) A simple method for improving protein solubility and long-term stability. J Am Chem Soc 126, 8933-9.

[3] Cavanagh J, Fairbrother WJ, Palmer III AG, Rance M, Skelton NJ (2007) Protein NMR spectrocopy: principles and practice. Elsevier Inc.

[4] Harris KS, Casey JL, Coley AM, Masciantonio R, Sabo JK, et al. (2005) Binding hot spot for invasion inhibitory molecules on *Plasmodium falciparum* apical membrane antigen 1. Infect Immun 73, 6981-9.

[5] Merutka G, Dyson HJ, Wright PE (1995) 'Random coil' ^1^H chemical shifts obtained as a function of temperature and trifluoroethanol concentration for the peptide series GGXGG. J Biomol NMR 5: 14-24.

[6] Yang S, Ad Bax (2010) SPARTA+: a modest improvement in empirical NMR chemical shift prediction by means of an artificial neural network. J Biomol NMR 48:13-22
